# Synthesis of Cu_x_O/Ag nanoparticles on exfoliated graphene: application for enhanced electrochemical detection of H_2_O_2_ in milk

**DOI:** 10.1038/s41598-023-33661-7

**Published:** 2023-04-24

**Authors:** Jie Song, Yating Wan, Chen Yang, Qiuju Deng, Yingde Cui, Zhihong Yan, Yi Liu

**Affiliations:** 1 School of Pharmaceutical and Chemical Engineering, Guangdong Pharmaceutical University, Zhongshan, 528400 China; 2 College of Pharmacy, Guangdong Pharmaceutical University, Guangzhou, 510000 China; 3 Guangdong Provincial Key Laboratory of Advanced Drug Delivery Systems and Guangdong Provincial Engineering Center of Topical Precise Drug Delivery System, The Center for Drug Research and Development, Guangdong Pharmaceutical University, Guangzhou, 510006 Guangdong China; 4 Guangzhou Vocational University of Science and Technology, Guangzhou, 510555 China

**Keywords:** Biomarkers, Materials science, Nanoscience and technology

## Abstract

In this paper, a novel composite is constructed as a non-enzymatic hydrogen peroxide (H_2_O_2_) sensor by liquid-phase exfoliation method, which is composed of copper oxide, cuprous oxide and silver nanoparticles doped few-layer-graphene (Cu_x_O/Ag@FLG). Its surface morphology and composition were characterized by scanning electron microscopy (SEM) and X-ray photo spectroscopy (XPS), and its H_2_O_2_ sensing performances include catalytic reduction and quantitative detection were studied with electrochemical methods. Our sensor had a high sensitivity of 174.5 μA mM^−1^ cm^−2^ (R^2^ = 0.9978) in an extremely wide range of concentrations from 10 μM to 100 mM, a fast response (about 5 s) and a low limit of detection (S/N = 3) of 2.13 μM. The sensor exhibits outstanding selectivity in the presence of various biological interference, such as dopamine, ascorbic acid, uric acid, citric acid, etc. In addition, the constructed sensor continued 95% current responsiveness after 1 month of storage further points to its long-term stability. Last but not least, it has a good recovery rate (90.12–102.00%) in milk sold on the open market, indicating that it has broad application possibilities in the food industry and biological medicine.

## Introduction

H_2_O_2_ is commonly used as a bleaching agent, disinfectant, and preservative in the food industry, owing to its strong oxidation resistance, good antibacterial and bactericidal properties^1^. H_2_O_2_ is not only one of the by-products of many classical enzymatic reactions, such as glucose oxidation, uric acid oxidation, oxalic acid oxidation, amino acid oxidation, glutamic acid, lysine oxidation, etc.^2^, but also plays a significant role as the primary messenger molecule in the cellular and redox metabolism^3^. It is worth noting that H_2_O_2_ has strong oxidizability, ingesting residual H_2_O_2_ in food will consume antioxidant substances in the body, accelerate the aging process of the human body, and reduce the resistance after ingestion. Excessive H_2_O_2_ in the body will produce a large number of OH·, which participates electron transfer and hydroxylation reaction in biochemical process, damage cells, induce gene mutation and various diseases, even cause human cell canceration^4^. In 2017, the list of carcinogens was announced by International Agency for Research on Cancer of the World Health Organization, and H_2_O_2_ appeared in the list of Class III carcinogens^5^. At present, it has been detected in the market that excessive amounts of H_2_O_2_ residues exist in prepackaged milk, beverages, soy products, aquatic products, chicken feet, etc., posing a serious threat to human health. Therefore, the newly revised National Food Safety Standard in China (GB 2760-2014) stipulates that H_2_O_2_ can be used as a food processing aid in various food processing processes, and the residual amount does not need to be limited, but it needs to be removed from the finished product. The Food and Drug Administration (FDA) requires H_2_O_2_ to be used as an antimicrobial agent in milk, with a residual amount not exceeding 0.05 wt% in 21 CFR 184.1366. In addition, it is also stipulated that H_2_O_2_ solution should be used in the sterilization of food packaging materials, and the residual amount of H_2_O_2_ in distilled water after packaging should be less than 0.5 parts per million (testing should be conducted immediately after packaging) in 21 CFR 178.1005. Therefore, sensitive detection of H_2_O_2_ is crucial for monitoring the quality of food and for applications of clinical, and biomedical.

Nowadays, the methods used in the detection of H_2_O_2_ include titration, spectroscopy, chromatography, chemiluminescence and electrochemical methods^6^. The Chinese national standard GB 5009. 226-2016 stipulates that the determination methods for H_2_O_2_ in food residues are iodometry and titanium salt colorimetry. The quantitative limit of the above two methods is 3 mg kg^−1^. These two methods are cumbersome, time-consuming, poor selectivity, and low accuracy, making it difficult to achieve trace detection. However, electrochemical method has the advantages of rapid response, rapid response, high-cost efficiency, simple operation, high selectivity and high sensitivity^7^. The first generation electrochemical H_2_O_2_ detector relies on the reaction of enzyme with hydrogen peroxide. This enzyme-based technology has been mature and can now provide high sensitivity, high selectivity and low background noise^8^. Nevertheless, the complex process of immobilizing the enzyme on the electrode, strict conditions to maintain the enzyme activity, and the lack of long-term stability and reproducibility limit the application of this technology. More importantly, almost all enzymes are made of proteins, which will hinder the electrochemical reaction on the surface of electrode^9^. Therefore, non-enzymatic electrochemical H_2_O_2_ detection have been developed to eliminate these shortcomings due to its faster and more effective electron transport^10^.

Enzyme-free H_2_O_2_ sensors generally prepared by precious metal nanoparticles (NPs) include Au, Ag, Pt and Pd NPs^11^, As a precious metal, Ag NPs have large specific surface area, good stability, good biocompatibility, excellent conductivity and electrocatalytic activity^12^. It is reported that Tran et al. have successfully developed a series of electrochemical sensors based on AgNPs for sensitive detection for glucose and H_2_O_2_^13–15^. But expensive cost, poor tolerance immensely limits their mercantile application. Consequently, it is urgent to develop low-cost, reliable and sensitive H_2_O_2_ electrochemical sensing materials. So far, Co_3_O_4_, Fe_3_O_4_, Cu_2_O, MnO_2_ have been successfully applied in H_2_O_2_ electrochemical sensors^16^. CuO and Cu_2_O have also been developed to prepare H_2_O_2_ sensors, which are inexpensive and strong stability, but with low sensitivity and narrow linear detection range. However, metal NPs have limited catalytic ability, because they are easily oxidized, and aggregative owing to the existence of van der Waals force between NPs^17^. In order to improve this problem, a suitable carrier is needed. At present, the substrates that have been widely studied are usually carbon materials, including ordered mesoporous carbon, carbon nanotubes, carbon nanofibers and graphene^18^. Among them, graphene has high specific surface area, good thermal stability, good conductivity, stability and high electron transfer ability, and its surface defects can anchor metal NPs, so it is an excellent carrier material^19^. Most of the existing related studies use graphene oxide prepared by Hummer’s method as carbon substrate to prepare metal nanoparticle composites. The preparation process is complex, and the distribution position and size of metal particles are uneven, which affects the sensing performance of the composites^20^. The liquid phase exfoliated method is low-cost, simple and controllable, green and environmentally friendly, and easy to large-scale production. It has been studied for use in Cd^2+^ and Pb^2+^ sensors, as well as flavomycin sensors^20,21^.

In this work, we have used water-in-oil emulsion as a stabilizing system to synthesis Cu_x_O/Ag@FLG composite by a simple ultrasonic exfoliation method for the first time. After a series of characterization, the morphology and structure of the composite were studied and modified on a glassy carbon electrode (GCE) to explore the detection of H_2_O_2_ performance. Compared with the reported Ag, CuO or Cu_2_O NPs for H_2_O_2_ sensors, the Cu_x_O/Ag@FLG/GCE H_2_O_2_ electrochemical sensor has the advantages of simple preparation, low cost and stable physicochemical properties. Our electrochemical experiments show the prepared sensor displays wide linear range, high sensitivity and selectivity toward the reduction of H_2_O_2_ with a low detection, and we also demonstrate it is successfully used to detect the concentration of H_2_O_2_ in milk. This study would offer a new routine for developing graphene-based electrochemical sensors for detecting H_2_O_2_.

## Experimental

### Materials

Graphite was purchased from Sinopharm Chemical Reagent Co.,Ltd (Shanghai, China), Tween 80, NH_3_·H_2_O purchased from Shanghai Yien Chemical Technology Co., Ltd (Shanghai, China), cyclohexane were purchased from Guangzhou Chemical Reagent Factory (Guangzhou, China), Span 80 was purchased from Shanghai yuanye Bio-Technology Co., Ltd (Shanghai, China), ethanol, CuSO_4_·5H_2_O, AgNO_3_, NaBH_4_, NaCl, FeCl_3_, glucose (Glu) and CaCl_2_ were purchased from Shanghai Aladdin Biochemical Technology Co., Ltd (Shanghai, China), MgCl_2_ were purchased from Damao Chemical Reagent Factory (Tianjin, China), hydrogen peroxide(H_2_O_2_) was purchased from Jiangsu Qiangsheng functional Chemistry Co., Ltd (Jiangsu, China), Ltd (Beijing, China), glycine (Gly), lysine (Lys) were purchased from Nine-Dinn Chemistry Co., Ltd. (Shanghai, China), dopamine (DA), uric acid (UA), citric acid (CA), lactic acid (LA), arginine (Arg), valine(Val) were purchased from Shanghai McLean Biochemical Technology Co., Ltd (Shanghai, China), polyvinylpyrrolidone k30 (PVP k30) was purchased from Shanghai bide Pharmaceutical Technology Co., Ltd (Shanghai, China).

### Synthesis of Cu_x_O/Ag@FLG composite

0.4 g graphite powder, 5 mL 3 wt% CuSO_4_ in 2 wt% PVP solution, 3 g Tween 80, 4 g Span 80, 5 mL anhydrous ethanol are added into 30 mL cyclohexane, stable emulsion can be formed through vortex mixing. Then, ultrasonic unit is used to exfoliate the above solution for 2 h, with 40 kHz ultrasonic power. After that, 0.057 g NaBH_4_ is added into the emulsion above and exfoliate the above solution for 2 h. Finally, the solid were carefully collected by filter to obtain Cu_x_O@FLG.

0.4 g Cu_x_O@FLG, 5 ml 3 wt% AgNO_3_ in 2 wt% PVP and 20% NH_3_·H_2_O mixing solution, 3 g Tween 80, 4 g Span 80, 5 mL anhydrous ethanol are added into 30 mL cyclohexane, stable emulsion can be formed through vortex mixing. Then, ultrasonic cleaner is used to exfoliate the above solution for 2 h. At the end, the solid were carefully collected by filter to obtain Cu_x_O/Ag@FLG.

### Fabrication of Cu_x_O/Ag@FLG/GCE electrodes

Before modification, bare GCE was polished with 0.05 μm alumina powder and ultrasonically cleaned with ethanol and water for 5 min. 5 μL of 1 mg mL^−1^ of well-dispersed Cu_x_O/Ag@FLG solution (include 0.05 wt% Chitosan and 0.5% acetic acid) was dropped to the washed GCE and dried under an infrared drying lamp for 10 min to prepare Cu_x_O/Ag@FLG/GCE. For comparison, we prepared Cu_x_O@FLG/GCE and Ag@FLG/GCE using the same method.

### Analytical procedure

JSM-7610F PLUS scanning electron microscope (SEM, JEOL, Tokyo, Japan), H-7650 transmission electron microscope (TEM, Hitachi, Tokyo, Japan), D/max-2200/PC X-ray diffractometer (XRD, Hitachi, Tokyo, Japan) and ESCALAB 250 X-ray Photoelectron Spectroscopy (XPS, Thermo Fisher Scientific, Massachusetts, America) were used to characterize the surface morphology and structure. Cyclic Voltammetry (CV), electrochemical impedance spectroscopy (EIS) and Chronoamperometry (i–t) were determined by the CHI660E electrochemical workstation (CH Instruments, Shanghai, China). Electrolyte was consisting of phosphoric acid buffer solution, the working electrode was modified GCE, the counter electrode was platinum sheet, the reference electrode was saturated calomel electrode (SCE). The i–t test was performed by adding H_2_O_2_ within a time interval of 40 s.

## Results and discussion

### Characterizations of morphology

**Figure 1:**
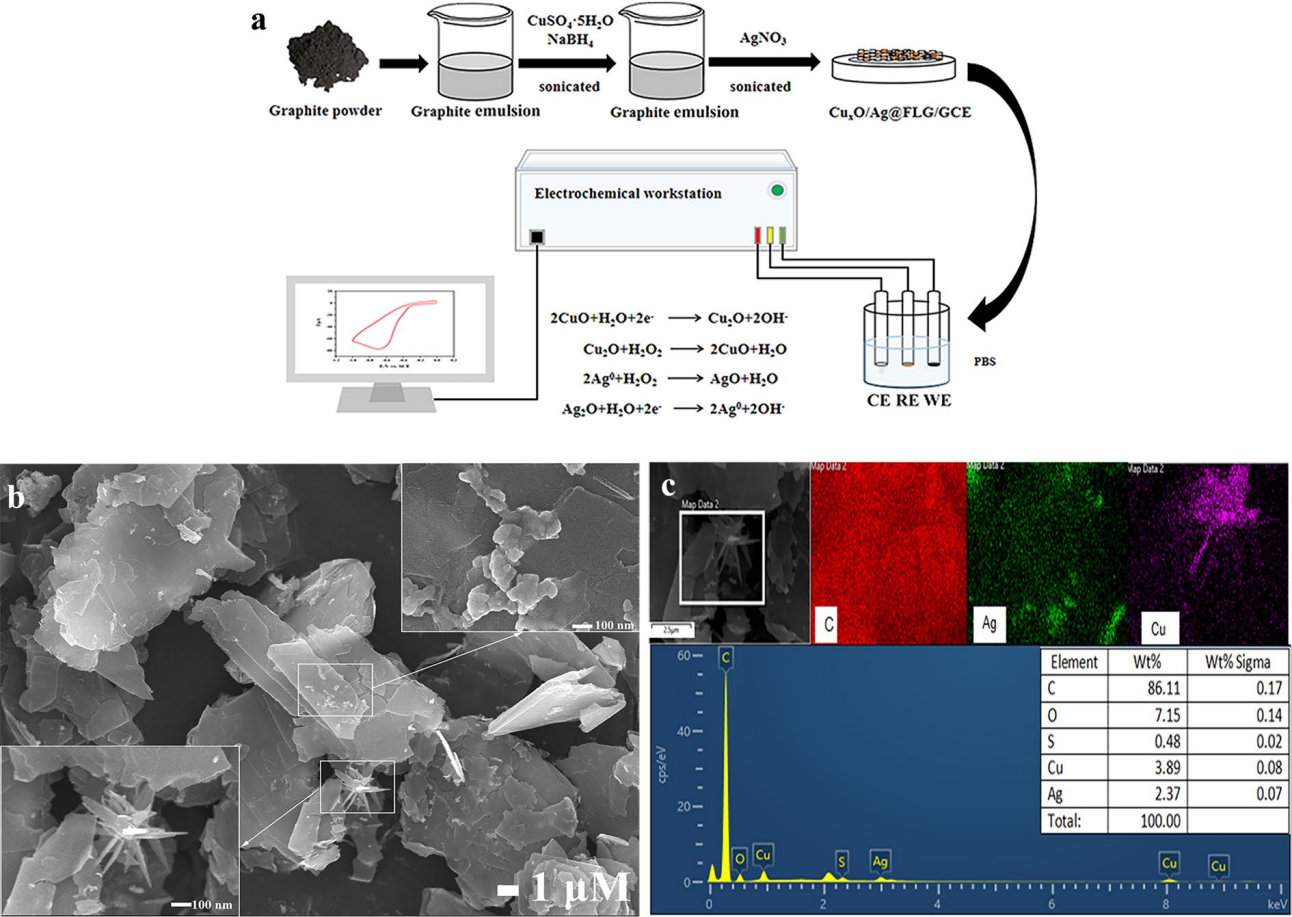
(**a**) preparation of Cu_x_O/Ag@FLG and mechanism of detecting H_2_O_2_. (**b**)SEM images and (**c**) EDS images of Cu_x_O/Ag@FLG.

The construction and detection principles of the Cu_x_O/Ag@FLG/GCE sensor are summarized in Fig. 1a. Next, the morphology of the material was characterized. The SEM image depicted in Fig. 1b shown that FLG with distinct crumples surface and large size were obtained, and there are lots of Ag nanoparticles and flower-like Cu_x_O nanosheet supported on the surface of crumble layers of exfoliated graphene. Furthermore, EDS images shown in Fig. 1c confirm the existence and homogenous distribution of C, O, Cu, Ag elements. Two-dimensional conductive network structure has been developed in Cu_x_O/Ag@FLG, which is conducive to the rapid transfer and conduction of electrons, so as to detect target analytes sensitively.

**Figure 2:**
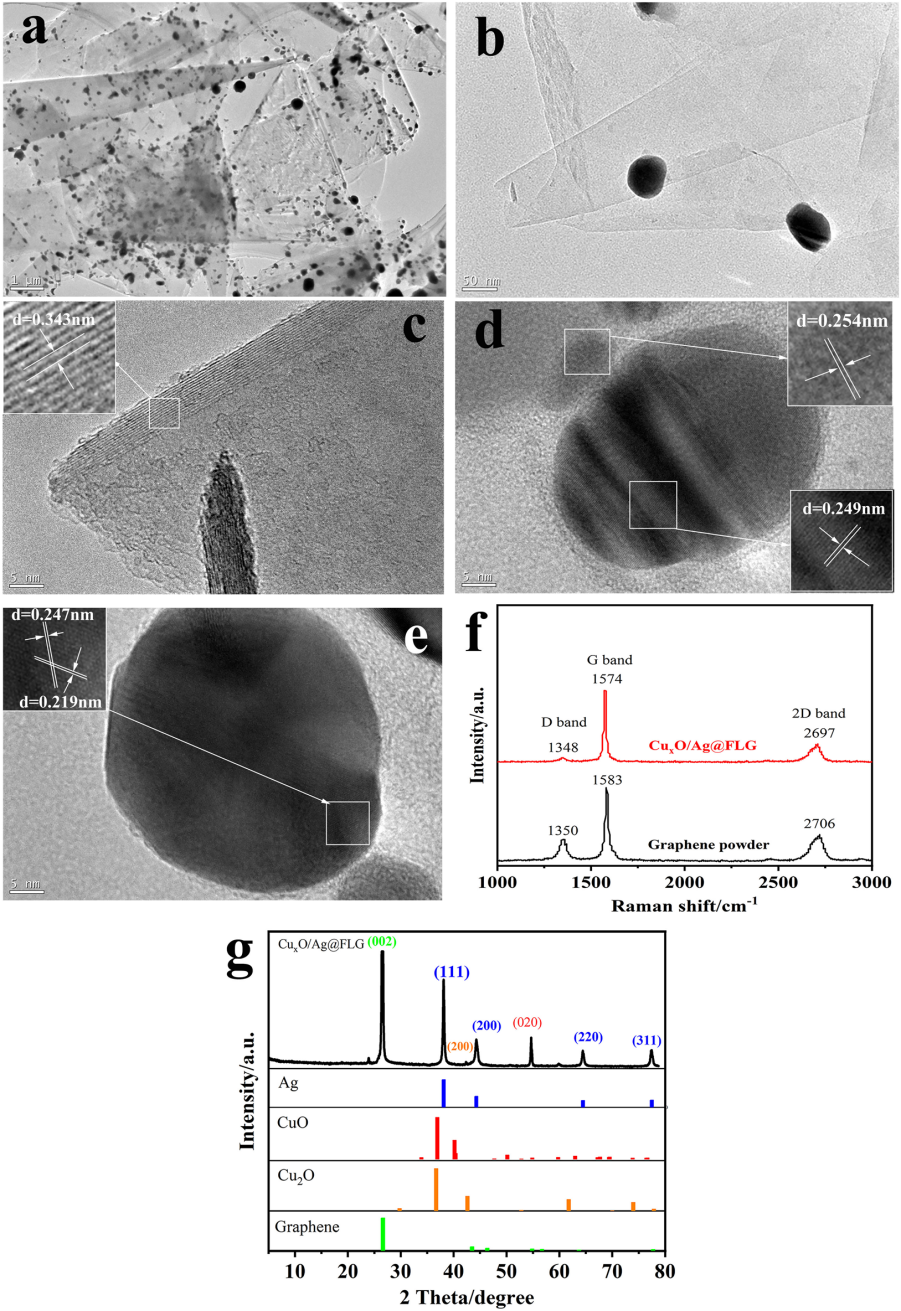
(**a**,**b**) TEM images, (**c**–**e**) high resolution TEM (HRTEM) images and (**f**) Raman spectra (**g**) XRD pattern of Cu_x_O/Ag@FLG.

As a supplement, the micromorphologies and microstructures of synthetic Cu_x_O/Ag@FLG investigated by TEM. As shown in Fig. 2a,b, the metal NPs were successfully loaded on the graphene sheet. The lattice spacing in Fig. 2c was 0.343 nm, belonging to the (002) crystal plane of graphene (JCPDS no. 41-1487). Interplanar distances of 0.249 nm and 0.247 nm indexed to the (002) crystal plane of CuO (JCPDS no. 45-0937), interplanar distance of 0.219 nm belongs to the (200) crystal plane of Cu_2_O (JCPDS no. 74-1230). The lattice spacing of 0.254 nm corresponds to the (111) crystal plane of Ag^0^ (JCPDS no. 04-0783) (Fig. 2d,e). In addition, the Raman spectrum compares the graphite powder raw material with the prepared material, as shown in Fig. 2f and Table 1. The peak position of the G peak and 2D peak move to the low frequency direction, and the half peak width of 2D peak decreases, and the I_2D_/I_G_ increases, which indicate the successful preparation of FLG^22^. Besides, the same trend as TEM images could be observed in XRD pattern. As can be seen from Fig. 2g, the strong peak at 26.38° is the characteristic peak of graphene’s (002) plane (JCPDS no. 41-1487). The peaks at 38.08°, 44.26°, 64.4°, and 77.46° can be assigned to (111), (200), (220) and (311) planes of Ag^0^ (JCPDS no. 04-0783). Other peaks at 42.33° and 53.54° respectively arise from (200) plane of Cu_2_O and (020) plane of CuO (JCPDS no. 74-1230 and JCPDS no. 45-0937).

**Table 1:** The Raman fitting data of graphite powder and Cu_x_O/Ag@FLG.

Materials	D band	G band	2D band	2D FWHM	I_D_/I_G_	I_2D_/I_G_
Graphite powder	1350	1583	2706	84.83	0.626	1.122
Cu_x_O/Ag@FLG	1348	1574	2697	81.15	0.204	4.779

### Characterizations of element binding states

**Figure 3:**
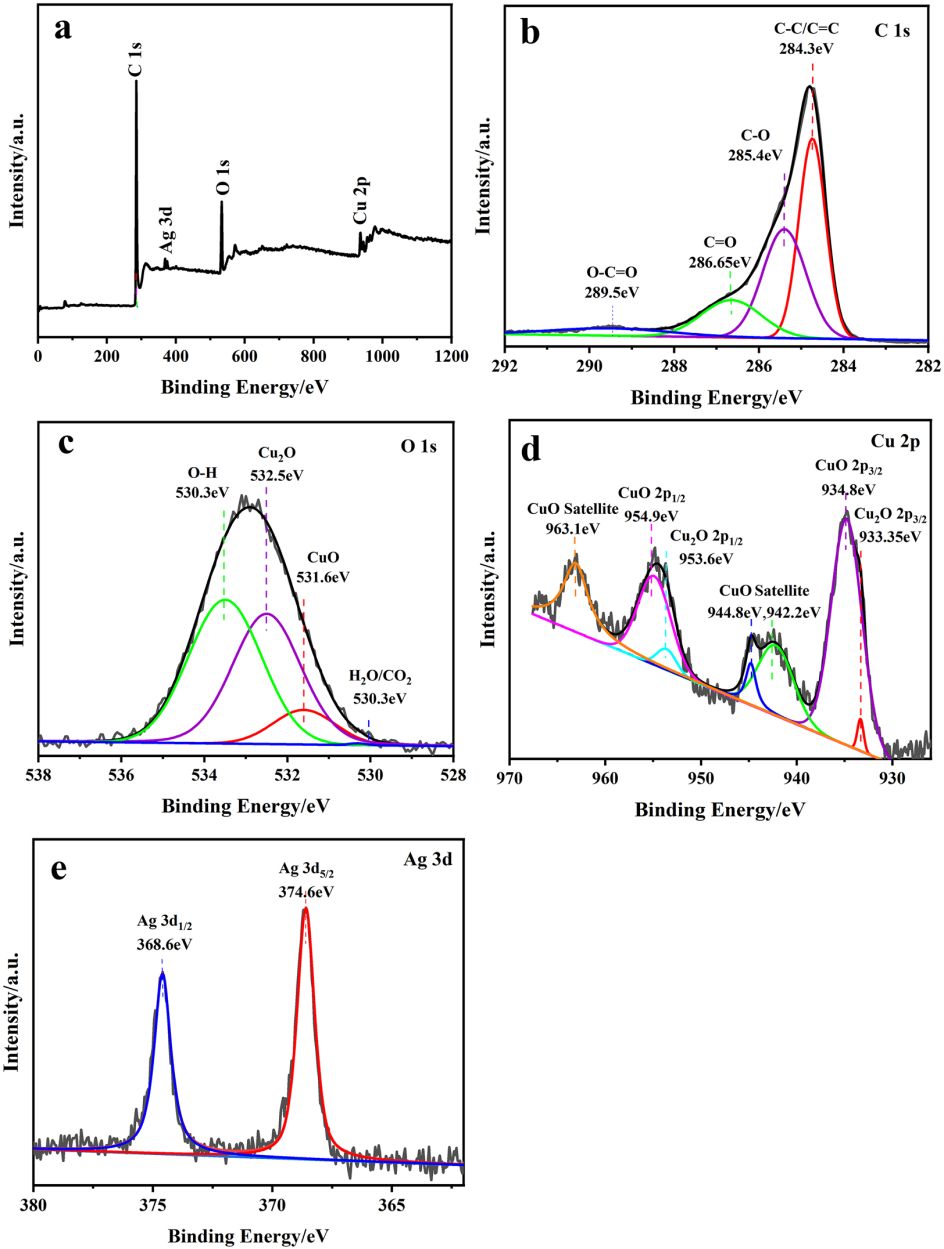
XPS spectra of Cu_x_O/Ag@FLG: (**a**) full survey spectrum. High-resolution spectra of (**b**) C 1s, (**c**) O 1s, (**d**) Cu 2p, and (**e**) Ag 3d, respectively.

Furthermore, the element composition and valence states of elements Cu_x_O/Ag@FLG nanocomposites were analyzed by XPS. The full scan XPS spectra of Cu_x_O/Ag@FLG showed signals corresponding to C 1s, O 1s, Cu 2p and Ag 3d (Fig. 3a). As exhibited in Fig. 3b, the peaks at 284.3, 285.4, 286.65 and 289.5 eV are attributed to C–C/C=C, C–O, C=O and O–C=O species. Compared to graphene oxide, the above C 1s XPS spectrum is more similar to reduced graphene oxide, because the peak intensity for C–C are significantly stronger than C–O and C=O^23^. Figure 3c indicated that the characteristic peaks at 531.6 eV, 532.5 eV which belong to lattice oxygen of Cu_2_O and CuO^24^, respectively, and the peaks at 533.5 eV, 530.3 eV are usually attributed to O in adsorbed –OH groups or carbonates^25^. The Cu 2p spectrum (Fig. 3d) was deconvoluted into seven peaks. The peaks at the binding energy of 933.35 eV of Cu 2p_3/2_ and 953.6 eV of Cu 2p_1/2_ are consistent with the representative spin–orbit of Cu^+^_._ At the same time, the peaks at the binding energy of 934.8 eV of Cu 2p_3/2_ and 954.9 eV of Cu 2p_1/2_ are characteristic peaks for Cu^2+^^26^. The peaks at 942.2, 944.8 and 963.1 eV are correspond to satellite peaks of Cu 2p in CuO^27^. The Ag 3d spectrum (Fig. 3e) was deconvoluted into two peaks, the peak of Ag 3d_5/2_ at 368.6 eV and the peak of Ag 3d_1/2_ at 374.6 eV are attributed to Ag^0^^28^.

### Electrochemical characterizations of the modified electrodes

**Figure 4:**
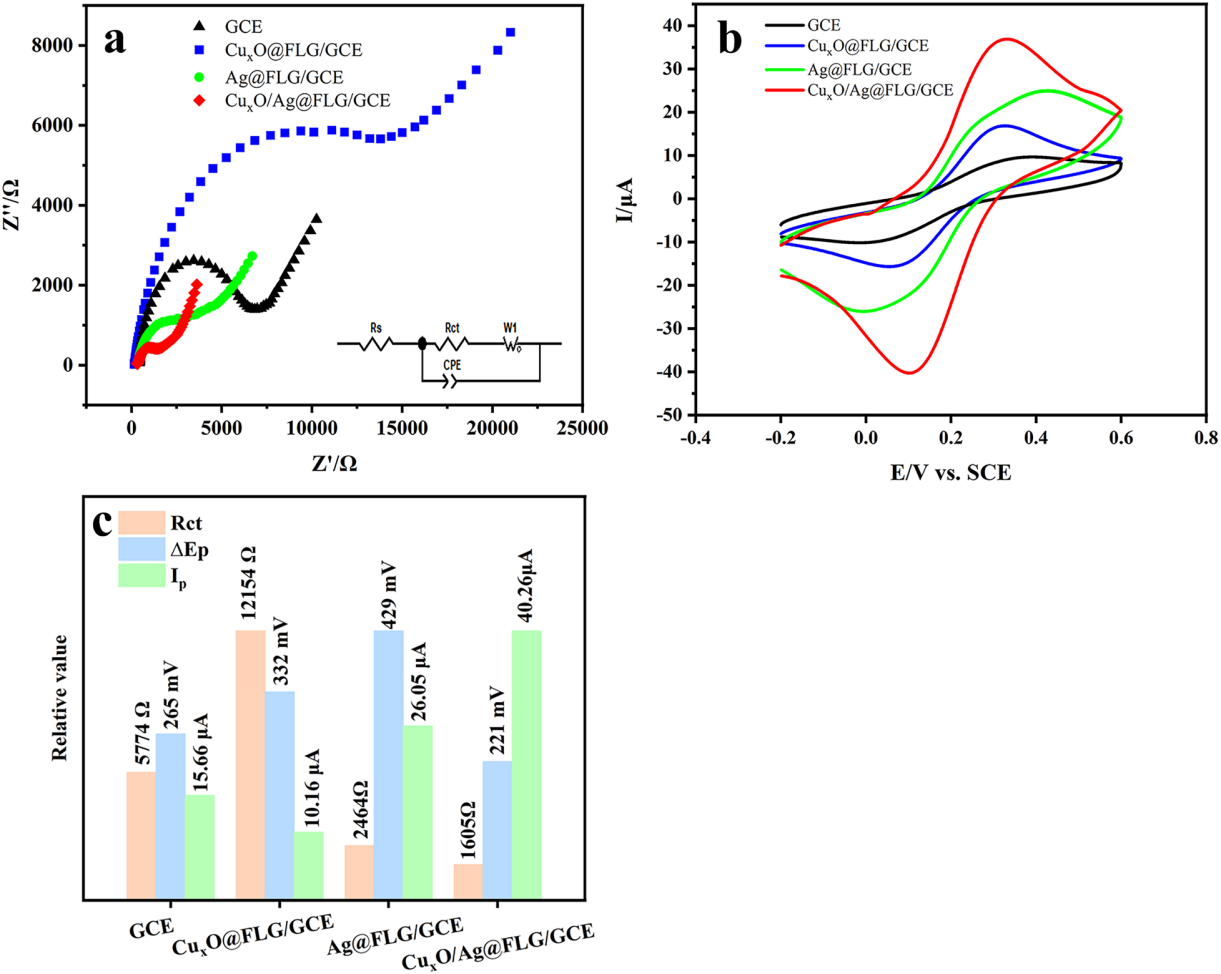
(**a**) EIS of bare GCE, Cu_x_O@FLG/GCE, Ag@FLG/GCE, Cu_x_O/Ag@FLG/GCE in 1 mM K_3_[Fe (CN)_6_]/1 mM K_4_[Fe (CN)_6_]/100 mM KCl solution 10^−1^–10^5^ Hz. The equivalent circuit is inserted; (**b**) CV of bare GCE, Cu_x_O@FLG/GCE, Ag@FLG/GCE, Cu_x_O/Ag@FLG/GCE in 1 mM K_3_[Fe (CN)_6_]/1 mM K_4_[Fe (CN)_6_]/100 mM KCl solution at a scanning speed of 50 mV·s^−1^; (**c**) the histogram of Rct, ΔE_p_ and I_p_ of different electrodes.

As depicted in Fig. 4, the interfacial characteristics of the modified electrodes were investigated by EIS. Generally, the diameter semicircle at high frequency represents the resistance to charge transfer (Rct)^29^. In Fig. 4a, compared with the Rct of bare GCE (5774 Ω), the Rct increases to 12,154 Ω after modified with Cu_x_O@FLG by reason of weak conductivity of Cu_2_O and CuO. However, the Rct decreases to 2464 Ω when modified with Ag@FLG, which indicates that Ag@FLG can promote electron transfer greatly. Furthermore, It is worth noting that Cu_x_O/Ag@FLG/GCE has the smallest Rct (1605 Ω), which illustrates its excellent electron transport ability owing to the large surface area and the potential synergism of the nanocomposites. Figure 4b further confirms the above inference peak-to-peak separation (ΔE_p_) of GCE, Cu_x_O@FLG/GCE, Ag@FLG/GCE and Cu_x_O/Ag@FLG/GCE are 265, 332, 429 and 221 mV respectively, indicating Cu_x_O/Ag@FLG can promote the electron transfer on the electrode surface to the most extent. The peak current of Cu_x_O/Ag@FLG/GCE is the largest, which is 3.98 times of GCE, 2.58 times of Cu_x_O@FLG/GCE and 1.55 times of Ag@FLG/GCE. It is clear that Ag@FLG/GCE has a larger peak current of redox peaks of [Fe(CN)_6_]^3−/4−^ couple, suggesting that the electrochemical probe of [Fe(CN)_6_]^3−/4−^ has good electron transfer at Ag@FLG/GCE due to its excellent electronic conductivity. However, on Cu_x_O@FLG/GCE, the redox peaks current of [Fe(CN)_6_]^3−/4−^ are much smaller, suggesting that the material of Cu_x_O@FLG is less effective for the electron transfer of the [Fe(CN)_6_]^3−/4−^ because of its inferior conductivity. It is interesting that when the Cu_x_O/Ag@FLG/GCE electrode was applied, the redox peak current was enhanced much more comparison with Ag@FLG, showing that the Cu_x_O/Ag@FLG/GCE nanocomposite has better performance for electron transfer of electrochemical probe than the single-component of Ag@FLG or Cu_x_O@FLG. Comparison histograms of Rct, ΔE_p_ and peak current (I_p_) of different electrodes are shown in Fig. 4c.

### Electrochemical detection of H_2_O_2_

**Figure 5:**
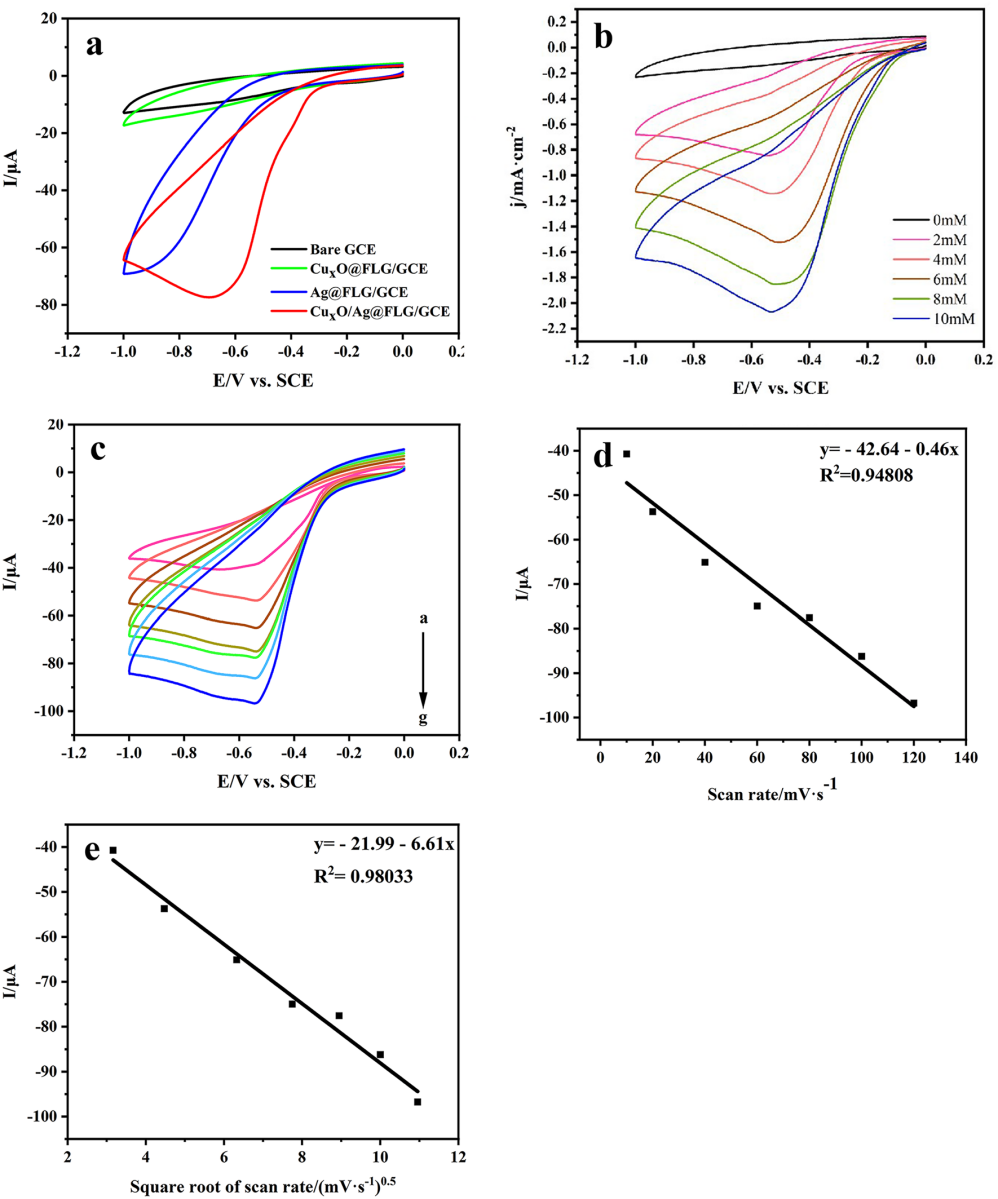
(**a**) CV of bare GCE, Cu_x_O@FLG/GCE, Ag@FLG/GCE, Cu_x_O/Ag@FLG/GCE in the presence of 4 mM H_2_O_2_; (**b**) CV curves of Cu_x_O/Ag@FLG/GCE in the presence of 0, 2, 4, 6, 8, 10 mM H_2_O_2_; (**c**) CV curves of Cu_x_O/Ag@FLG/GCE in the presence of 4 mM H_2_O_2_ at various scan rates; plots of cathodic peak current (I_p_) vs. (**d**) root of scan rate or (E) square root of scan rate (v^1/2^).

Electrochemical reduction of H_2_O_2_ by the synthesized composite was studied using CV method. Therefore, the CV curves are shown in Fig. 5 in N_2_-saturated 0.01 M PBS contained 4 mM H_2_O_2_ at a scan rate of 50 mV s^–1^. The reduction peak current enhancement of Cu_x_O@FLG/GCE is extremely weak (Fig. 5a). In addition, Ag@FLG show higher current response thanks to exceptional electrocatalytic activity of Ag NPs. CV curve of Cu_x_O/Ag@FLG/GCE has shown a lower reduction potential at − 0.65 V and higher current of 80 μA. Therefore, the Cu_x_O/Ag@FLG/GCE is a prospective electrochemical sensor for H_2_O_2_ detection. With the increase of H_2_O_2_ addition, the cathodic current increases linearly in Fig. 5b. Subsequently, the CV curves of Cu_x_O/Ag@FLG at varying scan rates (10–120 mV s^−1^) in 0.01 M PBS (pH 7.4) contained 4 mM H_2_O_2_ were investigated (Fig. 5c). Along with the square root of the scanning speed, the reduction peak current increases linearly, and correlation coefficients (R^2^) was fitted as 0.9803, which indicates that the diffusion-controlled electrochemical process (Fig. 5d,e)^30^.

### Mechanism for electrocatalytic reduction

The reaction mechanism of H_2_O_2_ electrocatalytic reduction of Cu_x_O/Ag@FLG nanocomposites is illustrated in the following chemical formula reduction. Primarily, CuO reduced to Cu_2_O, and Cu_2_O of the composite and that be reduced reacted with H_2_O_2_ to form CuO and H_2_O. Equations (1) and (2) can convey the reduction of H_2_O_2_ by Cu_x_O on Cu_x_O/Ag@FLG composite. Besides, Ag^0^ of Cu_x_O/Ag@FLG composite was oxidated to Ag_2_O with H_2_O_2_ was restored to H_2_O. Equations 3 can represent the reduction of H_2_O_2_ by Ag^0^ on Cu_x_O/Ag@FLG composite.

1 $${\text{2CuO}} + {\text{H}}_{{2}} {\text{O}} + {\text{2e}}^{ - } \to {\text{Cu}}_{{2}} {\text{O}} + {\text{2OH}}^{ - }$$

2 $${\text{Cu}}_{{2}} {\text{O}} + {\text{H}}_{{2}} {\text{O}}_{{2}} \to {\text{2CuO}} + {\text{H}}_{{2}} {\text{O}}$$

3 $${\text{2Ag}}^{0} + {\text{H}}_{{2}} {\text{O}}_{{2}} \to {\text{Ag}}_{{2}} {\text{O}} + {\text{H}}_{{2}} {\text{O}}$$

4 $${\text{Ag}}_{{2}} {\text{O}} + {\text{H}}_{{2}} {\text{O}} + {\text{2e}}^{ - } \to {\text{2Ag}}^{0} + {\text{2OH}}^{ - }$$

### Amperometric detection of H_2_O_2_ at Cu_x_O/Ag@FLG/GCE

**Figure 6:**
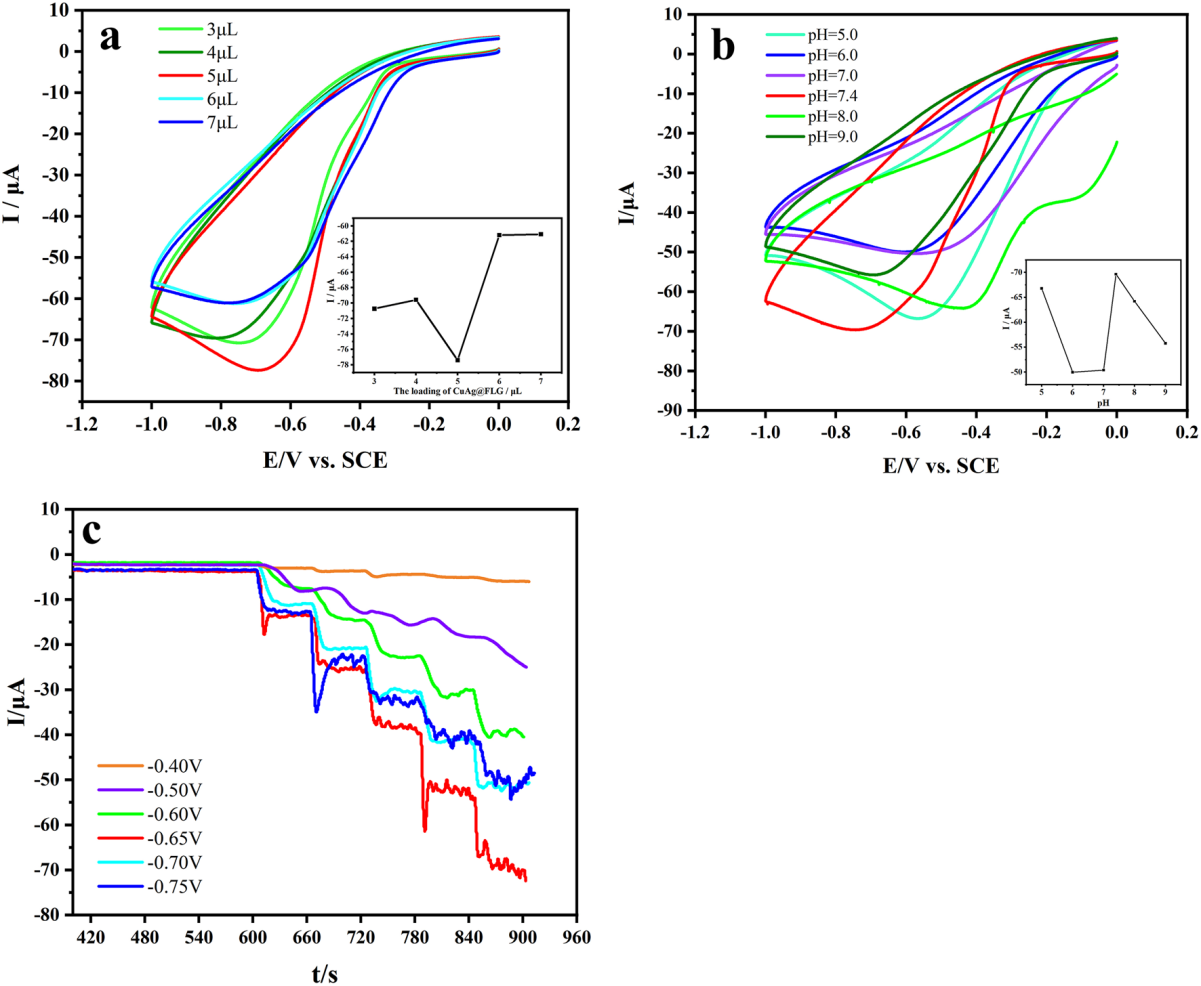
CVs at a scan rate of 0.05 V s^−1^ of Cu_x_O/Ag@FLG/GCE (**a**) with different volume of Cu_x_O/Ag@FLG in 0.01 M PBS (pH 7.4) containing 4.0 mM H_2_O_2_; (**b**)in PBS with different pH include 5.0, 6.0, 7.0, 7.4, 8.0, 9.0 containing 4 mM H_2_O_2_. (**c**) I–t curves of Cu_x_O/Ag@FLG/GCE at applied potentials with successive addition of H_2_O_2_ into 0.01 M PBS.

Then, the parameters for detecting H_2_O_2_ were optimized which include volume of catalyst ink, pH of supporting electrolyte and applied potential. The response current is the largest when the volume of ink was 5 µL (Fig. 6a). Furthermore, volume of 5 µL Cu_x_O/Ag@FLG composite on GCE is used for further studies. As shown in the Fig. 6b, CV curves was tested in PBS with different pH containing 4 mM H_2_O_2_, as a result, the maximum current response appears in PBS with pH 7.4. Therefore, PBS with pH 7.4 was selected as the electrolyte solution. The influence of different applied potentials on sensitivity was investigated by i–t method (Fig. 6c). The sensitivity was the highest at − 0.65 V. Hence, − 0.65 V is selected as the detection potential in the following studies.

**Figure 7:**
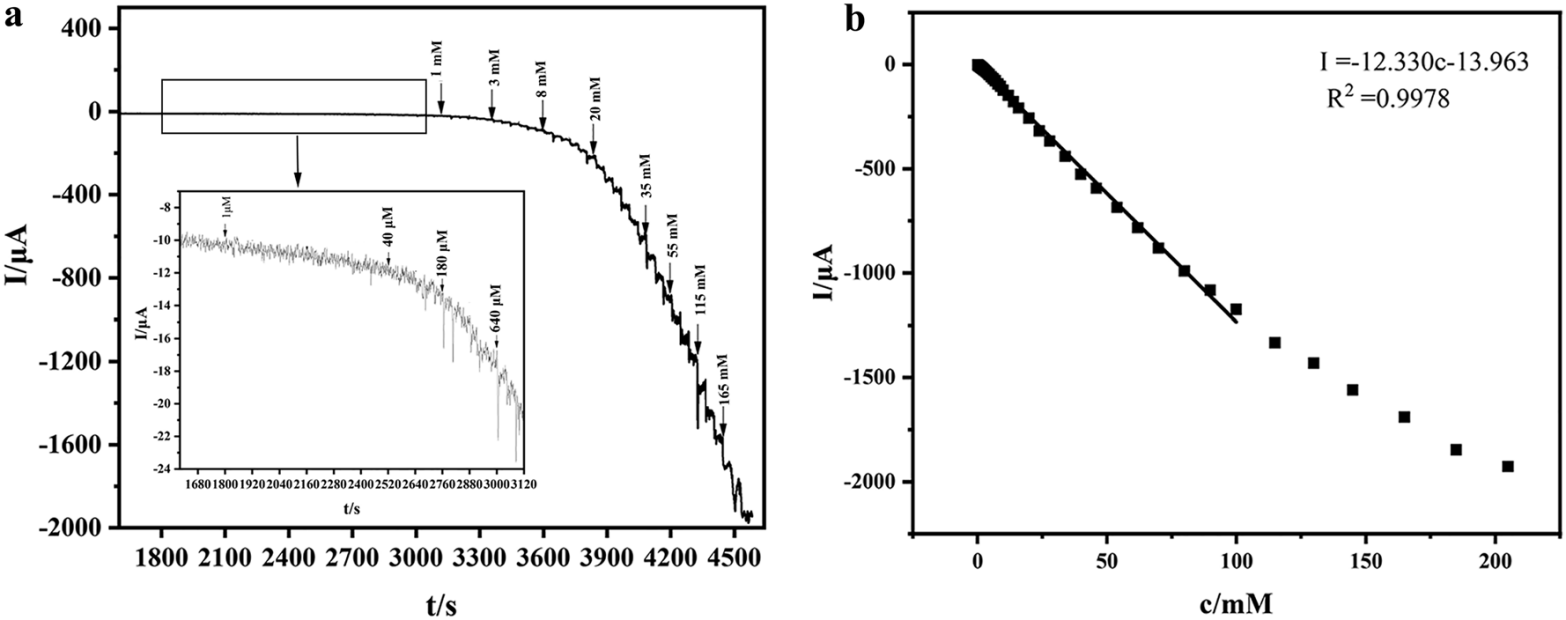
(**a**) I–t curve of the Cu_x_O/Ag@FLG/GCE for consecutive additions of H_2_O_2_ at − 0.65 V. The illustration shows the enlarged i–t curve in the low concentration area; (**b**) corresponding fitting curve.

Under the best conditions (5 μL Cu_x_O/Ag@FLG ink, 0.01 PBS with pH 7.4, − 0.65 V), i–t curve was plotted for Cu_x_O/Ag@FLG/GCE as H_2_O_2_ was added every 40 s (Fig. 7a). The response time of Cu_x_O/Ag@FLG/GCE to H_2_O_2_ is about 5 s. Meanwhile, the linear relationship between concentration of H_2_O_2_ and current is fitted (Fig. 7b), the current increases linearly with increasing concentration in the range of 10–100, 000 μM, the linear equation is I (μA) = − 12.330 c (c is the concentration of H_2_O_2_, mM) − 13.963, R^2^ = 0.9978, the detection limit is 2.13 μM (S/N = 3), and the sensitivity is 174.5 μA mM^−1^ cm^−2^. These are attributed to the large specific surface area of graphene sheet and the synergistic effect of graphene, Cu_x_O and Ag NPs in reducing H_2_O_2_ and amplifying signal.

**Figure 8:**
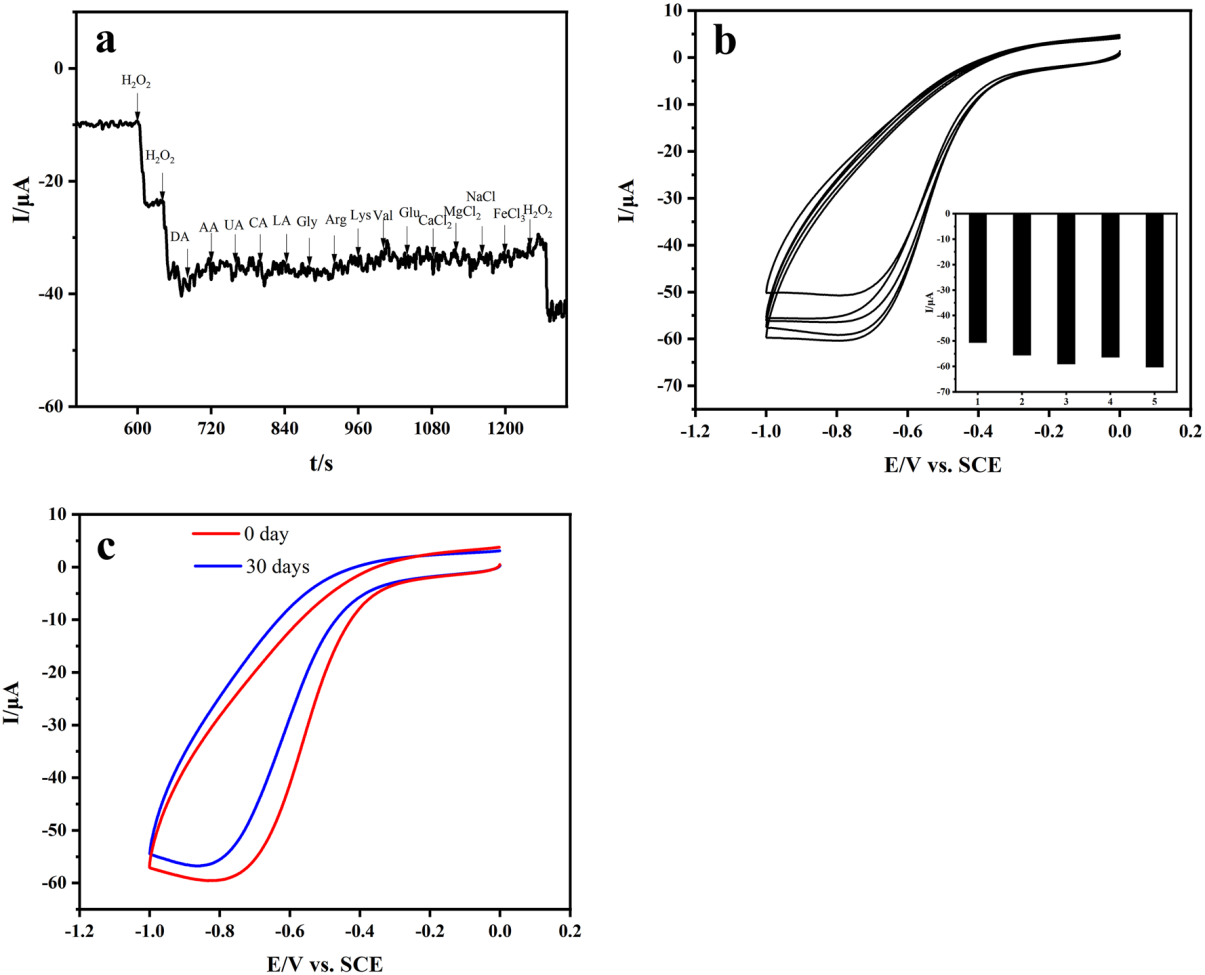
(**a**) Amperometric response recorded at Cu_x_O/Ag@FLG/GCE in 0.01 M PBS with the additions of H_2_O_2_ (1 mM), DA, AA, LA, Gly, UA, Arg, Lys, CA, Val, Glu, CaCl_2_, MgCl_2_, NaCl, FeCl_3_; (**b**) CV curves of Cu_x_O/Ag@FLG/GCE in 0.01 M PBS contained 4 mM H_2_O_2_ at five equally prepared Cu_x_O/Ag@FLG/GCE sensors; (**c**) CV curves of the Cu_x_O/Ag@FLG/GCE in PBS contained 4 mM H_2_O_2_ tested at 1st day and 30th day.

Figure 8a demonstrates the i–t curve of the Cu_x_O/Ag@FLG/GCE sensor after addition of 1 mM H_2_O_2_, 1 mM Val, DA, AA, UA, CA, LA, Gly, Arg, Lys, Glu, CaCl_2_, MgCl_2_, NaCl, FeCl_3_, and a second injection 1 mM H_2_O_2_. After adding interferent, the current response to H_2_O_2_ on Cu_x_O/Ag@FLG/GCE at the detection potential is very small, which indicates that the response of the sensor to H_2_O_2_ is almost not affected in the presence of other compounds, Cu_x_O/Ag@FLG/GCE shows high selectivity. Long-term stability and reproducibility are momentous factors which evaluate the performance of sensors. Five Cu_x_O/Ag@FLG/GCE electrodes were prepared respectively, and the relative standard deviation (RSD) of current response in the same concentration of H_2_O_2_ was 3.69%, which presents a good repeatability (Fig. 8b). After storing at room temperature for 1 month, the reaction of the prepared electrode to H_2_O_2_ decreased to about 95% (Fig. 8c), which illustrates that it has long-term stability. In a word, our research makes it clear that Cu_x_O/Ag@FLG/GCE is reliable as an electrochemical sensor of H_2_O_2_. At the same time, it has the potentiality to detect H_2_O_2_ in real samples in complex environment.

The comparison of Cu_x_O/Ag@FLG/GCE sensor with other H_2_O_2_ sensors containing CuO, Cu_2_O and Ag is listed in Table 2. Significantly, as-synthesized sensor in this study has a wider detection range and a lower detection limit. Compared with other reported electrochemical sensors for H_2_O_2_ detection, the Cu_x_O/Ag@FLG/GCE electrode has a significant advantage in terms of reaction time and sensitivity. Furthermore, the production and application of the obtained Cu_x_O/Ag@FLG/GCE electrode are inexpensive, simple and easy to control, resulting in a potential perspective for convenient, fast, sensitive and cost-effective detection of H_2_O_2._

**Table 2:** Comparison of H_2_O_2_ sensors related to CuO, Cu_2_O and Ag in different literature.

Modified material	Detection limit (μM)	Linear range (μM)	Sensitivity (μA μM^−1^ cm^−2^)	References
CuO/g-C_3_N_4_/GCE	0.31	0.5–50	3.327	^31^
CuO-SWCNT-PDDA/GCE	20.8	300–7800	4.396	^32^
Cu_2_O–rGO	21.7	30–12,800	0.0195	^33^
Cu_2_O/TiO_2_/Ti	90.5	500–8000	0.412	^34^
CQDs/octahedral Cu_2_O/GCE	8.4	20–4300	0.000298	^35^
Ag NPs/3DG/GCE	14.9	30–16,210	1.094	^36^
Ag NPs/N-G/GCE	1.2	100–126,400	0.0446	^37^
Ag/Cu_2_O/ITO	0.1	500–30,000	0.0502	^38^
Cu_x_O/Ag@FLG/GCE	2.13	10–100,000	0.1745	This work

### Detection of H_2_O_2_ in milk sample

In addition, the H_2_O_2_ concentration in milk samples were determined to further evaluate the availability and practical application of the Cu_x_O/Ag@FLG/GCE. To reduce the sample matrix effect, milk from two different brands was centrifuged for 15 min at 7, 000 rpm, then diluted 10 times with deionized water and filtered with 0.22 μM microporous membrane filtration to obtain milk solution. Finally, the obtained recovery of H_2_O_2_ was calculated using the standard addition method. Amperometric curve was detected at − 0.65 V under successive adding the milk sample, which were displayed in Fig S1. As shown in Table 3, the recovery for H_2_O_2_ in the sample was 90.12–102.00%.

**Table 3:** Actual sample analysis of CuxO/Ag@FLG/GCE in milk (n = 3).

Real sample	Added (mM)	Found (mM)	Recovery (%)	Titrimetric method (mM)
Milk 1	0.050	0.051	102.00	0.053
	0.175	0.167	95.56	0.165
	0.425	0.383	90.12	0.432
Milk 2	0.050	0.048	96.00	0.056
	0.175	0.172	98.29	0.184
	0.425	0.401	94.35	0.384

## Conclusions

To summaries, Cu_x_O/Ag@FLG sensor has been prepared successfully by ultrasonic exfoliation method. Cu_x_O/Ag@FLG/GCE electrode has shown excellent catalytic reduction performance for H_2_O_2_, such as wide linearity range (10–100, 000 μM), low detection limit (2.13 μM), high sensitivity (174.5 μA mM^−1^ cm^−2^) and long-term stability. In addition, the prepared sensor can detect H_2_O_2_ in milk samples, which makes it possible for quality control in food industry, suggesting that as-synthetic Cu_x_O/Ag@FLG has a broad application prospect in electrochemical sensors.

## Supplementary Information

Supplementary Information.

## Data Availability

The datasets generated during and/or analysed during the current study are available from the corresponding author on reasonable request.
