# Supplementary material for: Synthesis of CuxO/Ag nanoparticles on exfoliated graphene: application for enhanced electrochemical detection of H2O2 in milk
Source: Sci Rep. 2023 Apr 24;13:6640. doi: 10.1038/s41598-023-33661-7 (PMC10126113; doi:10.1038/s41598-023-33661-7)
Supplement: Supplementary file 1 — Supplementary Information. [file 41598_2023_33661_MOESM1_ESM.docx]

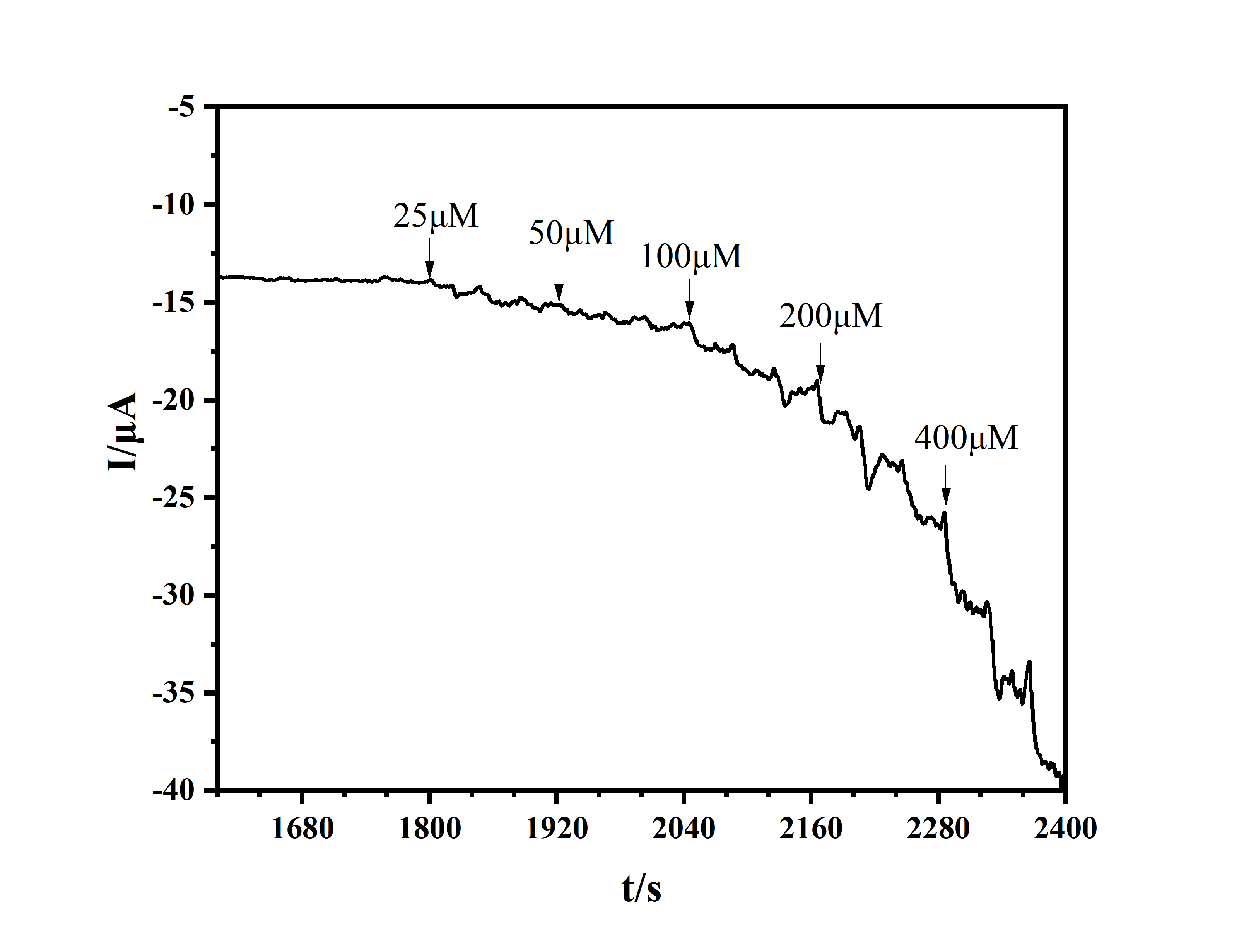


**a**


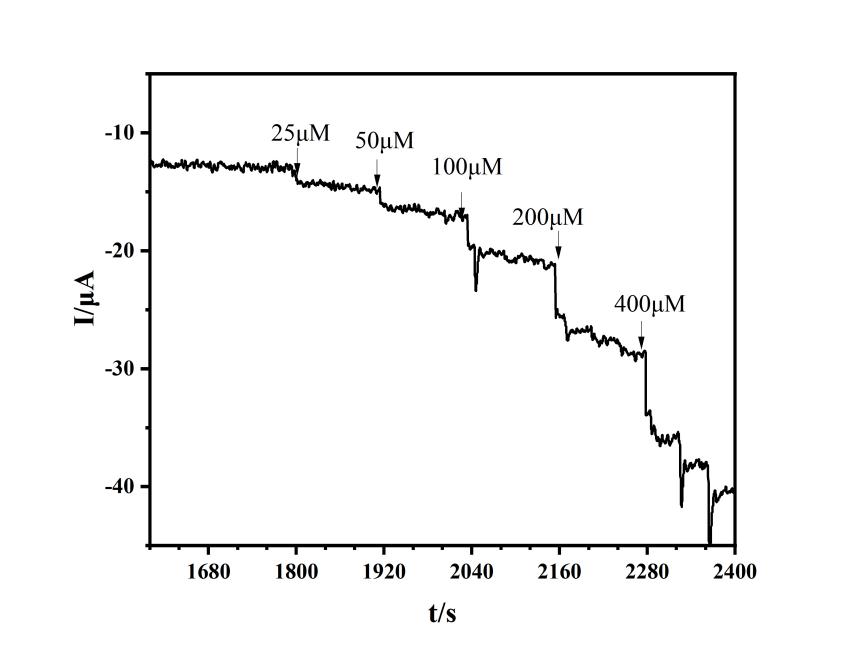


**b**

Figure S1. I-t cure of Cu_x_O/Ag@FLG/GCE electrode to successive addition of (a) milk 1 and (b) milk 2 samples into PBS at applied potential of -0.65 V.
